# Cost-Effectiveness of Pembrolizumab Plus Chemotherapy as First-Line Therapy for Advanced Oesophageal Cancer

**DOI:** 10.3389/fphar.2022.881787

**Published:** 2022-05-30

**Authors:** Meiyu Wu, Shuxia Qin, Liting Wang, Chongqing Tan, Ye Peng, Xiaohui Zeng, Xia Luo, Lidan Yi, Xiaomin Wan

**Affiliations:** ^1^ Department of Pharmacy, The Second Xiangya Hospital, Central South University, Changsha, China; ^2^ Institute of Clinical Pharmacy, Central South University, Changsha, China; ^3^ PET Imaging Center, The Second Xiangya Hospital, Central South University, Changsha, China

**Keywords:** pembrolizumab, oesophageal cancer, cost-effectiveness, markov model, chemotherapy

## Abstract

**Objective:** Pembrolizumab plus chemotherapy is recommended as the first-line treatment for advanced oesophageal cancer. The objective of this study is to evaluate the cost-effectiveness of pembrolizumab plus chemotherapy as first-line therapy for advanced oesophageal cancer from the healthcare system perspective in China.

**Methods:** Based on the KEYNOTE-590 trial, a Markov model was constructed to estimate the cost and effectiveness of pembrolizumab plus chemotherapy and placebo plus chemotherapy, respectively. Total costs, life years (LYs), quality-adjusted life years (QALYs), and incremental cost-effectiveness ratios (ICERs) were calculated. One-way, probabilistic sensitivity analyses (PSA), and subgroup analyses were adapted to test the model robustness.

**Result:** Compared with the placebo group, pembrolizumab group obtained an additional 1.05 QALY, but the cost was also increased by $121,478.76. The ICER was $115,391.84 per QALY gained, which was higher than the willingness-to-pay (WTP) of $31,304.31. The results of One-way sensitivity analyses showed that the ICER was sensitive to the hazard ratio of PFS and per cycle cost of pembrolizumab. At a WTP threshold of $31,304.31, the probability of pembrolizumab plus chemotherapy being cost-effective was 0%.

**Conclusion:** From the perspective of China healthcare system, pembrolizumab plus chemotherapy as first-line treatment is not cost-effective for patients with advanced oesophageal cancer compared with placebo plus chemotherapy.

## Introduction

Oesophageal cancer ranks seventh in incidence and sixth in mortality worldwide. Eastern Asia occupies the highest incidence rate, partly because of the heavy burden in China (Sung et al., 2021). In 2019, the number of new cases and deaths of esophageal cancer in China are about 278,120 and 257,315 respectively. The total number of disability-adjusted life years (DALYs) is 5,759,997, which accounted for 8.6% of all cancer DALYs (Institute for Health Metrics and Evaluation, 2019; Qiu et al., 2021). The histologic subtypes of oesophageal cancer are divided into squamous cell carcinoma, adenocarcinoma, and other subtypes. In China, about 90% of patients with esophageal cancer are diagnosed with squamous cell carcinoma, and most patients are diagnosed at advanced stages (Chen et al., 2016; Li et al., 2017; Abnet et al., 2018). The 5-year survival rate of patients with advanced oesophageal cancer remains at a low level (Enomoto et al., 2021).

At present, fluorouracil combined with platinum, or paclitaxel combined with platinum, are the most recommended first-line treatment options for patients with advanced or metastatic oesophageal cancer (Muro et al., 2019; National Comprehensive Cancer Network, 2022). However, these treatments have little effect on improving overall survival (OS) in patients with advanced esophageal cancer. Compared with chemotherapy alone, the combination of immune checkpoint inhibitors (ICIs) and chemotherapy showed more effective antitumor activity in several studies (Gandhi et al., 2018; Cortes et al., 2020; Sun et al., 2021).

Pembrolizumab is a humanized monoclonal antibody that blocks the interaction between PD-1 and its ligands, PD-L1 and PD-L2. The phase Ⅲ KEYNOTE-590 trial evaluated the efficacy and safety of pembrolizumab plus chemotherapy (pembrolizumab group) compare with placebo plus chemotherapy (placebo group) as first-line treatment in advanced oesophageal cancer and Siewert type 1 gastro-oesophageal junction cancer (Sun et al., 2021). In that study, 749 patients were randomized to the pembrolizumab group or placebo group and were followed up for a median of 22·6 months. The result showed that compared with chemotherapy alone, pembrolizumab plus chemotherapy prolonged median OS [12.4 months vs. 9.8 months; hazard ratio (HR) 0.64] and median progression-free survival (PFS; 6.3 months vs. 5.8 months; HR 0.65).

For patients with unresectable, metastatic oesophageal cancer, the combination of pembrolizumab could be considered as the first-line treatment (Sun et al., 2021). However, the high cost of pembrolizumab may increase the economic burden of society. This study aimed to evaluate the cost-effectiveness of Pembrolizumab plus chemotherapy as first-line therapy for advanced oesophageal cancer from the perspective of the Chinese healthcare system based on KEYNOTE-590 trial data.

## Methods

### Model Structure

**FIGURE 1:**
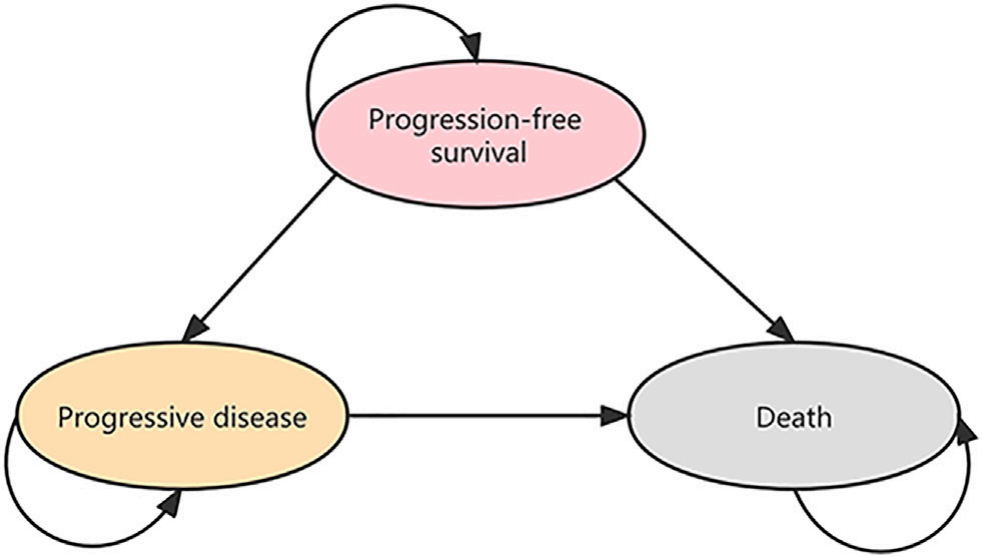
Model Structure for cost–effectiveness analysis.

We constructed a Markov cohort model to compare two first-line among patients with oesophageal cancer: 1) pembrolizumab, 5-fluorouracil, and cisplatin; and 2) placebo, 5-fluorouracil, and cisplatin. The model contained three mutually exclusive health states: PFS, progressive disease (PD), and death (Figure 1). All patients were in state PFS at the beginning and the patients could maintain the health state of the previous cycle or enter another health state in each cycle. We assumed that patients would receive second-line treatment after disease progression. The analysis used a lifetime horizon and the cycle length was 3 weeks. Costs and benefits were discounted at an annual rate of 3% (Sanders et al., 2016), and the half-cycle correction was used. Total costs, life years (LYs), quality-adjusted life years (QALYs), and incremental cost-effectiveness ratios (ICERs) were the primary outputs in the model. According to the recommendation of the World Health Organization (WHO), we applied 3 times per capita gross domestic product (GDP) of China as the willingness-to-pay (WTP) threshold, to judge whether pembrolizumab combined chemotherapy is cost-effective compared with chemotherapy alone ($31,304.31) (The World Bank, 2022). Model establishment and data analysis were performed in the R software (R version 4.0.5; http://www.r-project.org).

### Simulated Population

The characteristics of the simulated population are similar to the patients of the KEYNOTE-590 trial, which enrolled patients aged 18 years or older with previously untreated, locally advanced, unresectable oesophageal cancer or Siewert type 1 gastro-oesophageal junction adenocarcinoma. We assumed that the weight and height of the simulated population were 60 kg and 1.64 m, respectively, meaning a body surface area (BSA) of 1.21 (Zhang et al., 2021).

### Transition Probability

The estimation of the transition probability between different health states is mainly based on the KEYNOTE-590 trial. We obtained points on the PFS and OS curves through the GetData Graph Digitizer software (version 2.26; http://getdata-graph-digitizer.com) and then reconstructed the individual patient data (IPD) using R software according to the algorithm proposed by Guyot et al. (2012). Exponential, Weibull, log-normal, log-logistic and gompertz distribution were used to fit these data. Log-logistic distribution was selected as the best fit distribution because of its minimum Akaike information criterion (AIC) value. Natural death probabilities at different ages were also included, which were derived from the Chinese life tables (Word health Organization, 2022).

### Cost and Utility

In this study, we only considered the cost of drugs, routine follow-up, supportive care, treatment-related grade 3 or worse adverse events (AEs). In the first-line treatment, the usage and dosage of drugs are as follows: pembrolizumab 200 mg once every 3 weeks for up to 35 cycles, 5-fluorouracil 800 mg/m^2^ on days 1–5 plus cisplatin 80 mg/m^2^ on day 1 once every 3 weeks for up to 6 cycles. After disease progressed, patients who received subsequent anticancer therapy were 43% in the pembrolizumab group and 47% in the placebo group, respectively (Sun et al., 2021). We assumed that patients who received subsequent anticancer therapy all used paclitaxel monotherapy as second-line treatment in the model. The prices of drugs were obtained from The Second Xiangya Hospital of Central South University, and the rest of the costs were derived from previously published studies (Chongqing et al., 2014; Bai et al., 2017; Yang et al., 2021; Zhang et al., 2021). The costs of AEs were calculated by multiplying the incidence of AEs by the costs of managing the AEs per event. All costs were converted to 2020 United States dollars (USD) using the local Consumer Price Index ($1 = ¥6.9) (National Bureau of Statistics, 2021). The utility values of health states were from a published study, which assigned 0.74to PFS and 0.58 to PD (Cai et al., 2021) (Table1).

**TABLE 1:** Model parameters: baseline values and ranges.

Parameter	Baseline Value	Lower	Upper	Source
HR of OS between pembrolizumab and placebo groups	0.73	0.58	0.88	Sun et al. (2021)
HR of PFS between pembrolizumab and placebo groups	0.65	0.52	0.78	Sun et al. (2021)
Drug cost per cycle, $				
Pembrolizumab	5625.75	2812.87	8438.62	Local medical data
5-Fluorouracil	256.67	205.34	308.01	Local medical data
Cisplatin	17.52	14.01	21.02	Local medical data
Paclitaxel	351.81	281.44	422.17	Local medical data
Utility				
PFS	0.74	0.59	0.89	Cai et al. (2021)
PD	0.58	0.46	0.70	Cai et al. (2021)
Administration cost per cycle, $				
Routine follow-up	55.18	44.14	66.21	Zhang et al. (2021)
Supportive care	125.47	100.37	150.56	Zhang et al. (2021)
Treatment-related AEs, $				
Nausea	71.00	56.80	85.20	Yang et al. (2021)
Anaemia	73.68	58.94	88.42	Chongqing et al. (2014)
Decreased neutrophil count	466.00	372.80	559.20	Bai et al. (2017)
Neutropenia	466.00	372.80	559.20	Bai et al. (2017)
Decreased white blood cells	466.00	372.80	559.20	Bai et al. (2017)
Discount rate, %	3	0	5	Sanders et al. (2016)

HR, hazard ratio; OS, overall survival; PFS, progression-free survival; PD, progressed disease.

### Sensitivity Analysis

To evaluate the robustness of the model, one-way and probabilistic sensitivity analyses (PSA) were performed in this study. One-way sensitivity analyses is to observe the influence of different parameters on ICER by using the maximum and minimum values of the parameter. In this research, pembrolizumab fluctuated within ± 50% of the current price value, and other parameters changed within 95% confidence interval or ± 20% of the base value (Table1). The discount rate ranged between 0 and 5%. In the PSA, a 1000 times Monte Carlo simulation was performed by randomly generating different parameter values according to the specific distribution. Our study assumed that the utility parameter conformed to beta distribution and the cost parameter conformed to gamma distribution (Briggs et al., 2012). In addition, the threshold analysis for cycle cost of Pembrolizumab was performed.

## Result

### Base Case Analysis

The base-case analyses showed that patients in the pembrolizumab group had 5.36 LYs and 3.37 QALYs for $135,890.90. In the placebo group, patients had 3.71 LYs and 2.32 QALYs for $14,412.14. Compared with the placebo group, the mean incremental effect and cost were 1.05 QALYs and $121,478.76 for the pembrolizumab group. ICER was calculated by dividing the incremental cost by the incremental effect and was estimated to be $115,391.84 per QALY gained (Table2). At the WTP threshold of $31,304.31 in China, pembrolizumab plus chemotherapy as the first-line therapy for advanced oesophageal cancer was not a cost-effective strategy compared with placebo-chemotherapy.

**TABLE 2:** Base case results.

Results	Pembrolizumab group	Placebo group	Incremental
LYs	5.36	3.71	1.65
QALY	3.37	2.32	1.05
Cost, $	135,890.90	14,412.14	121,478.76
Cost Per LY gained, $	—	—	73,640.00
Cost Per QALY gained, $	—	—	115,391.84

LY, life year; QALY, quality-adjusted life year.

### Sensitivity Analysis

**FIGURE 2:**
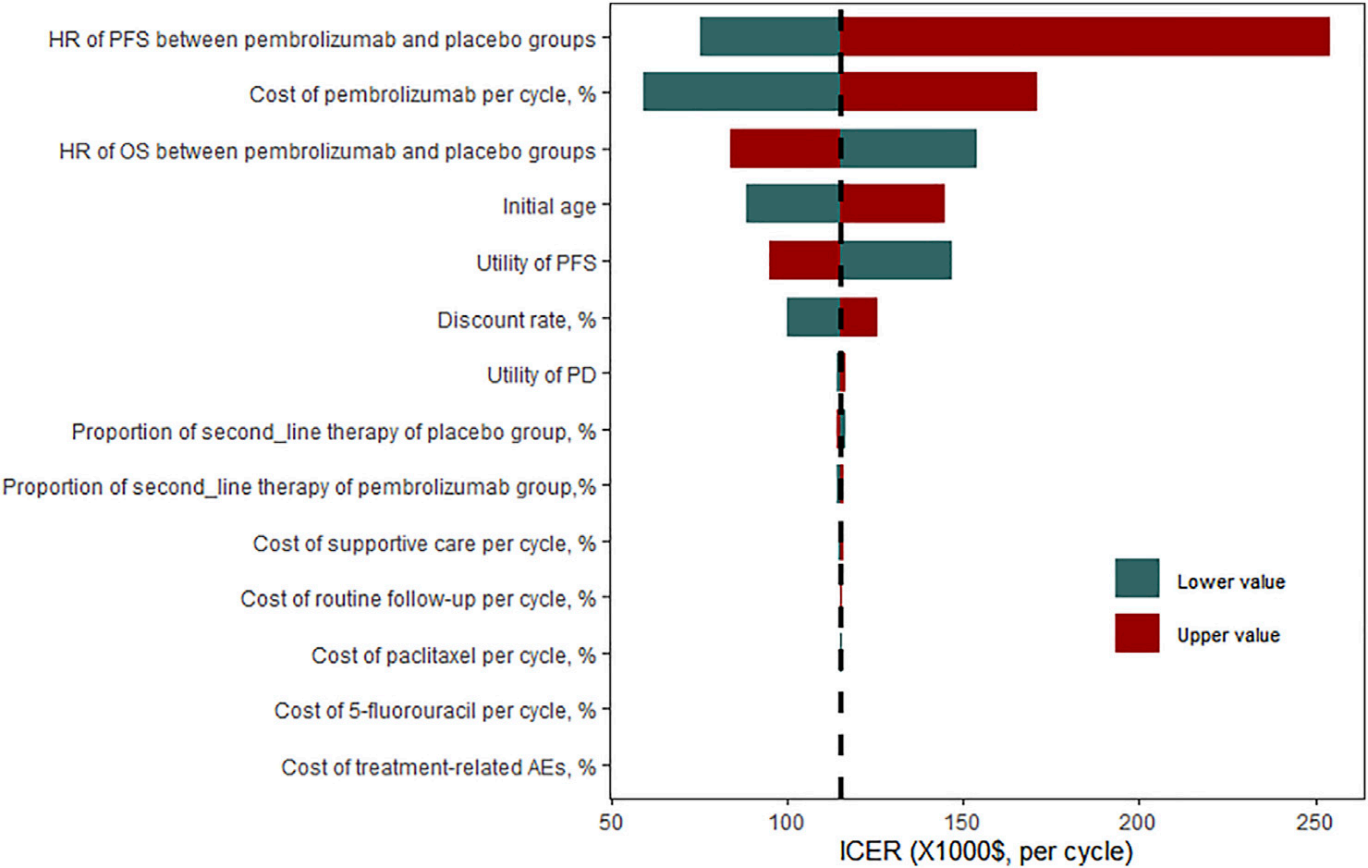
One-way sensitivity analysis for pembrolizumab versus placebo group. HR, hazard ratio; OS overall survival; PFS, progression-free survival; ICER, incremental cost-effectiveness ratio.

The results of the one-way sensitivity analyses demonstrated that the ICER was most sensitive to HR of PFS between pembrolizumab and placebo groups. The ICER was as high as $254,146.49 when the HR of PFS was 0.78, which far exceeded the WTP threshold of $31,304.31 per QALY gained in China. The cost of pembrolizumab per cycle, HR of OS, initial age of the patient cohort, and utility of PFS also had a significant impact on the model results. If the cost of pembrolizumab per cycle was reduced by 50%, the ICER was decreased to $59,482.61. However, all results indicated that pembrolizumab combined chemotherapy was not cost-effective compared with chemotherapy alone, with a WTP of $31,304.31 (Figure2). To make the ICER below $31,304.31 per QALY, the cost of pembrolizumab per cycle should be reduced by 74% (see Supplementary Table S2 in supplementary material).

**FIGURE 3:**
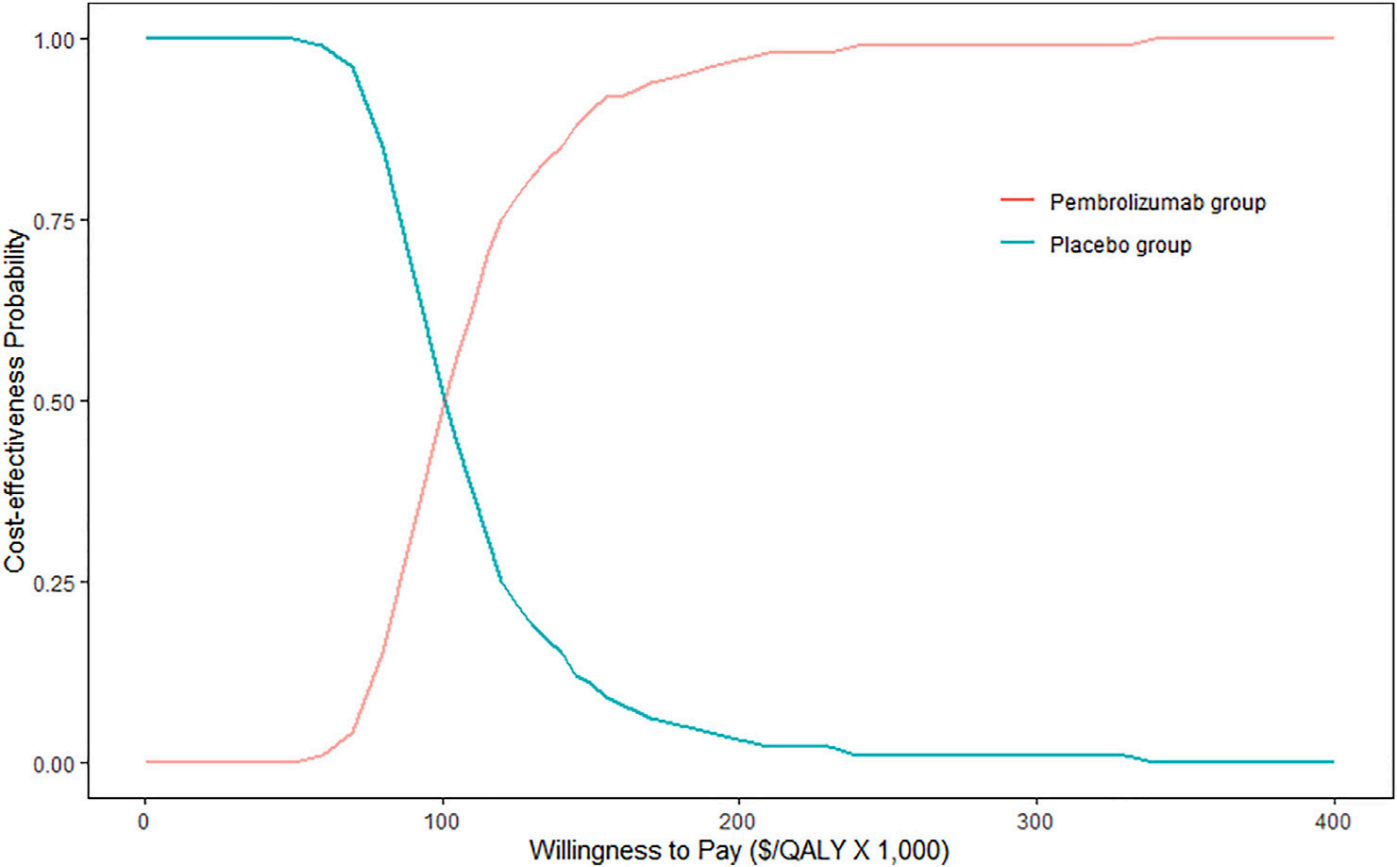
Cost-effectiveness acceptability curve. QALY, quality-adjusted life year.

Based on the Monte Carlo PSA, the probability of pembrolizumab plus chemotherapy being a cost-effective regime for patients with advanced esophageal cancer would be 50% if the WTP threshold was $100,500 per QALY gained. As the WTP threshold increases, the greater the probability that pembrolizumab plus chemotherapy would be cost-effective. When the WTP threshold was set at $340,000, the probability of pembrolizumab plus chemotherapy being cost-effective is 100% (Figure3).

### Subgroup Analysis

We performed subgroup analysis based on histology and PD-L1 status. In the subgroup analysis, the incremental cost and incremental QALY of patients with PD-L1 combined positive score (CPS) of 10 or more were $126,693.97 and 1.56, respectively. Resulting in an ICER of $81,236.42 per QALY gained, which was the lowest in all groups, but still higher than $31,304.31. More details are shown in Table3.

**TABLE 3:** Results of subgroup analysis.

Subgroup	Estimated OS HR	Estimated PFS HR	Incremental cost, $	Incremental QALYs	ICER, $/QALY
oesophageal squamous cell carcinoma	0.72	0.65	115,781.43	1.02	113,966.51
PD-L1 CPS of 10 or more	0.62	0.51	126,693.97	1.56	81,236.42

HR, hazard ratio; OS, overall survival; PFS, progression-free survival; QALY, quality-adjusted life-year; ICER, incremental cost-effectiveness ratio; CPS, combined positive score.

## Discussion

Base on the results of our results, the ICER of pembrolizumab plus chemotherapy for patients with advanced oesophageal cancer as first-line treatment in China is $115,391.84 per QALY gained, which was above the WTP threshold of $31,304.31 per QALY, indicating that pembrolizumab plus chemotherapy was not a cost-effective regime compared with placebo plus chemotherapy.

In September 2021, pembrolizumab was approved by the National Medical Products Administration (NMPA) to combine chemotherapy for the first-line treatment of patients with unresectable, locally advanced or metastatic oesophageal cancer, or gastro-oesophageal junction cancer, which means that pembrolizumab had become the first and only PD-1 monoclonal antibody approved for the first-line treatment of oesophageal cancer in China (National Medical Products Administration, 2022). However, pembrolizumab with higher price than a series of Chinese domestic ICIs (Camrelizumab: $424.35/200 mg, Toripalimab: $1088.04/240 mg, Sintilimab: $412.03/100 mg) (DRUGDATAEXPY, 2022). In the one-way sensitivity analyses, the cost of pembrolizumab per cycle played a very important role in affecting ICER. The cycle cost of pembrolizumab ranged from $2812.87 to $8438.62, the ICER varied from $59,482.61 to $171,301.30, which was still above $31,304.31.

But it does not mean that we can only choose chemotherapy alone with low safety and effectiveness. Clinically, the choice of treatment scheme needs to consider a variety of factors. In order to reduce the economic pressure of cancer patients and make the limited resources meet the needs of more people, China has rolled out many preferential policies related to anti-tumor drugs in recent years. For example, the price of camrelizumab per 200 mg has dropped from $2,802 in 2020 to $424.35 in 2021. Therefore, it is expected to reduce the price of pembrolizumab and improve its cost-effectiveness. Pembrolizumab plus chemotherapy would be cost-effective when the cycle cost of pembrolizumab is reduced to $1332 (74%), with the ICER of $30,996.13. The effectiveness of pembrolizumab had the greatest impact on ICER, the HR of PFS ranged from 0.52 to 0.78, the ICER varied from $75,487.06 to $254,146.49. Indeed, in the subgroup analysis, patients with PD-L1 CPS of 10 or more had the lowest HR and generated the lowest ICER. It seems that they can benefit most from the regimen of pembrolizumab plus chemotherapy. Value-based drug pricing would benefit the sustainability of the health care system and promote drug development. Our result shows that when the cost of pembrolizumab per cycle was halved, the ICER was above the $31,304.31 per QALY, which was not cost-effective. If the cost of pembrolizumab per cycle decreased by 74%, pembrolizumab would be cost-effective as a first-line strategy for advanced oesophageal cancer. In addition, selecting patients with PD-L1 CPS of 10 or more for treatment could improve the pharmacoeconomic profile of pembrolizumab. Reducing prices through negotiations on the trade-off between drug prices and coverage may be an appropriate and effective way to improve the cost-effectiveness.

At present, there are few studies on the pharmacoeconomic evaluation of oesophageal cancer. To our knowledge, this is the first cost-effectiveness analysis of pembrolizumab plus chemotherapy as first-line therapy for advanced oesophageal cancer. One recent study evaluated the cost-effectiveness of camrelizumab plus chemotherapy as first-line therapy for advanced or metastatic esophageal squamous cell carcinoma in China (Zhang et al., 2021). Camrelizumab combined with paclitaxel and cisplatin is recommended by the Chinese Society of Clinical Oncology (CSCO) Guidelines for the Diagnosis and Treatment of Esophageal Cancer in 2021 as the first-line treatment of advanced or metastatic esophageal squamous cell carcinoma (Guidelines Working Committee of Chinese society of clinical oncology, 2021). This study showed that the ICER of camrelizumab plus chemotherapy versus placebo plus chemotherapy was $46,671.10 per QALY gained, which was higher than the WTP threshold of $31,498.70 per QALY gained. Therefore camrelizumab plus chemotherapy is not a cost-effective strategy as the first-line treatment for advanced or metastatic esophageal squamous from the perspective of the Chinese healthcare system, which was basically consistent with our results. Another study is the cost-effectiveness analysis of nivolumab as the second-line treatment from the perspective of Chinese society (Zhang et al., 2020). The results of this study demonstrated that the ICER was $136,709.35 per QALY gained, and nivolumab is not a cost-effective treatment option based on the WTP threshold of $29,306.43 per QALY gained. On the basis of previous studies, although ICIs can prolong the OS of patients with advanced oesophageal cancer, ICIs are still not cost-effective due to the high price, whether it is a first-line treatment or second-line treatment.

Several limitations in our study must be acknowledged. First, the results of this study are obtained by modeling, which might not completely reflect the real world. However, we tested several distributions and selected the best fitting (log-logistic), which could reduce the inaccuracy of the model. Second, the utility values in the model were derived from previously published studies, which might not accurately reflect the utility of the KEYNOTE-590 trial. Although one-way sensitivity analyses showed that the utility used in the model had an impact on ICER, the ICER was still far above the WTP threshold of $31304.31, whether increasing or decreasing the utility. Third, in this model, we only consider paclitaxel as a second-line treatment. In reality, we should choose a suitable second-line treatment according to the actual situation. Luckily, the cost of second-line had a minor influence on economic outcomes. Fourth, we did not consider the cost of grade 1–2 AEs, but the results were not sensitive to the cost of treatment-related AEs based on the one-way sensitivity analyses.

## Conclusion

Based on our study, at the WTP threshold of $31,304.31 per QALY gained, pembrolizumab plus chemotherapy as first-line therapy is not a cost-effective regime for patients with advanced oesophageal cancer compared with placebo plus chemotherapy from the perspective of the Chinese healthcare system. However, the cost-effectiveness of pembrolizumab plus chemotherapy for patients with advanced oesophageal cancer is expected to improve as the price of pembrolizumab decreases.

## Data Availability

The original contributions presented in the study are included in the article/Supplementary Material, further inquiries can be directed to the corresponding author.
