# Supplementary material for: Cost-Effectiveness of Pembrolizumab Plus Chemotherapy as First-Line Therapy for Advanced Oesophageal Cancer
Source: Front Pharmacol. 2022 May 30;13:881787. doi: 10.3389/fphar.2022.881787 (PMC9197184; doi:10.3389/fphar.2022.881787)
Supplement: Supplementary file 1 [file Table1.DOCX]

Table S1. The results of goodness-of-fit

| Parametric survival models | AIC | |
| --- | --- | --- |
|  | Pembrolizumab group | Placebo group |
| **Overall survival** |  |  |
| Exponential | 2041.576 | 2242.540 |
| Weibull | 2028.544 | 2217.093 |
| Log-normal | 2017.552 | 2222.091 |
| Log-logistic | 2010.561 | 2205.896 |
| Gompertz | 2041.762 | 2235.512 |
| **Progression-free survival** |  |  |
| Exponential | 1966.190 | 1977.189 |
| Weibull | 1950.289 | 1927.401 |
| Log-normal | 1906.703 | 1918.959 |
| Log-logistic | 1903.900 | 1905.444 |
| Gompertz | 1968.033 | 1966.206 |
| **Log-logistic parameters** |  |  |
| Scale(λ) for OS | 0.014475 | 0.015156 |
| shape(γ) foe OS | 1.658448 | 1.829282 |
| Scale(λ) for PFS | 0.026662 | 0.030171 |
| shape(γ) foe PFS | 1.900869 | 2.163687 |

AIC, Akaike information criterion; OS, Overall survival; PFS, Progression-free survival


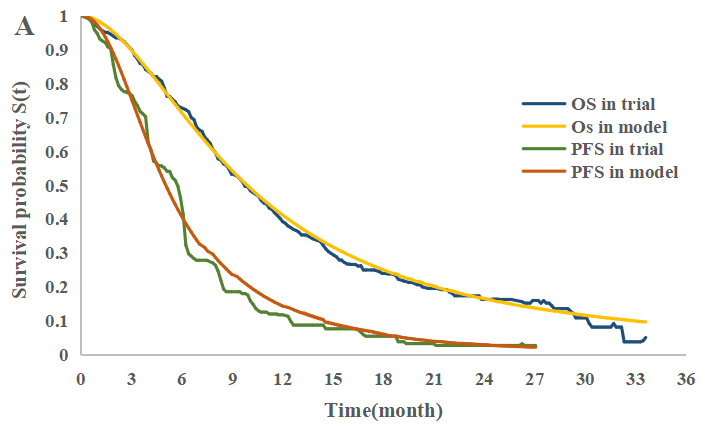

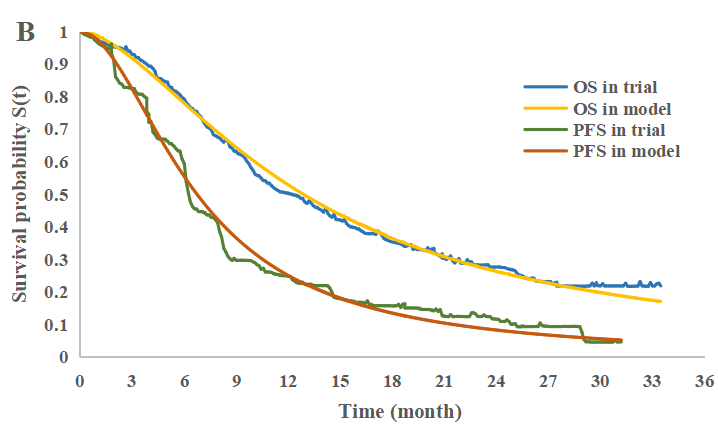


Figure S1. Estimated Log-logistic survival curves for the KEYNOTE-590 trial. (A) The placebo group, (B) the pembrolizumab group. OS, Overall survival; PFS, Progression-free survival.

Table S2. Threshold analysis for cycle cost of pembrolizumab

| Cost of pembrolizumab, $/cycle | ICER, $/QALY |
| --- | --- |
| 2812.87 (50% cost) | 59,482.61 |
| 1332.75 (26% cost) | 30,996.13 |
| 841.25 (15% cost) | 20,864.33 |

Abbreviations: ICER, incremental cost-effectiveness ratio.
